# Application of sex-specific single-nucleotide polymorphism filters in genome-wide association data

**DOI:** 10.1186/1753-6561-3-s7-s57

**Published:** 2009-12-15

**Authors:** Hua Ling, Kurt Hetrick, Joan E Bailey-Wilson, Elizabeth W Pugh

**Affiliations:** 1 Center for Inherited Disease Research, Institute of Genetic Medicine, Johns Hopkins University School of Medicine, Baltimore, Maryland 21224, USA; 2 Inherited Disease Research Branch, National Human Genome Research Institute, National Institutes of Health, Baltimore, Maryland 21224, USA

## Abstract

We explored five sex-specific quality control filters in North American Rheumatoid Arthritis Consortium's Illumina 550 k datasets. Three X chromosome and three autosomal single-nucleotide polymorphisms flagged by sex quality control filters were missed by filters of call rate at 95% and Hardy-Weinberg equilibrium at 10^-6^. We applied a subset of these sex-specific quality control filters to eight chromosomes in the Framingham Heart Study samples genotyped by Affymetrix 500 k SNP arrays, and identified another two single-nucleotide polymorphisms that failed to be picked up by the above global filters.

## Background

The advent of genome-wide association study (GWAS) arrays with hundreds of thousands of markers relegates manual review of genotyping data to, at best, a small subset of the markers, which often are the single-nucleotide polymorphisms (SNPs) of interest after analysis. SNP filters such as call frequency, minor allele frequency (MAF), Hardy-Weinberg equilibrium (HWE), replicate concordance, and mendelian errors have been widely implemented to identify problematic SNPs. For example, the SNP thresholds used by Plenge et al. [1] for the rheumatoid arthritis (RA) study that includes the North American Rheumatoid Arthritis Consortium (NARAC) samples were call frequency >95%, MAF>1%, and HWE *p*-value>10^-6 ^(the NARAC and Swedish Epidemiologic Investigation of Rheumatoid Arthritis (EIRA) data sets were filtered separately) [1]. Such global SNP filters are effective in removing SNPs with clustering problems, thus reducing the large number of highly significant associations, and lowering genomic control value lambda toward expectation. However, manual review of SNP plots suggests that some good SNPs are deleted and some bad ones remain. In particular, we have observed SNPs on the X chromosome for which the male and female samples cluster differently on Illumina GWAS arrays. The raw and normalized intensity plots for these SNPs exhibit different clusters by sex. Calls for one sex may be missing or assigned to an incorrect genotype. Also, Illumina's Beadstudio calling algorithm ignores sex in clustering, and will make illogical calls, e.g., heterozygous calls for males on the X chromosome or genotype calls for females on the Y chromosome. We have noted sex differences for a small number of pseudo-autosomal and autosomal markers as well.

Such SNPs, where data is differentially missing, miscalled, or both by sex may be of particular concern for diseases with differences in risk by sex, such as RA. In this analysis, we report on sex-specific differences in SNP call rates and genotype frequencies in the NARAC and Framingham Heart Study (FHS) data as released in Genetic Analysis Workshop 16 (GAW16).

## Methods

Data were extracted from NARAC and FHS data files for GAW16 and analyzed using PLINK 1.04 [2]. In the NARAC dataset, DNA samples from 2,062 individuals (868 cases and 1,194 controls, 569 males and 1493 females) were genotyped on Illumina 550 k arrays. For the FHS dataset, 6,848 individuals were genotyped on Affymetrix GeneChip^® ^Human Mapping 500 k Array Set. We used the 6,808 of FHS samples with a specified sex (3,105 males and 3,703 females).

The overall sample and SNP statistics including observed proportion of heterozygotes (PropHet), call rate, and MAF were calculated using PLINK. We explored three approaches to identifying X chromosome SNPs that may be called differently by sex. The simplest were the proportion of male heterozygote calls and the absolute value of the difference in the call rates for males and females. The other, more statistical approach was to code all samples as female (because PLINK drops male heterozygote genotypes on X), use sex as the phenotype, and test whether the proportion of missing data was associated with sex (--test-missing option in PLINK [2]). We used four approaches for autosomes: the absolute value of the differences in the call rates and proportion of heterozygotes in all samples, the test of missing data by sex, and a test of allelic association with sex among the controls (--test-missing and --assoc option in PLINK [2]). SAS and Spotfire DecisionSite 9.1.1 [3] were used for additional calculations and for plots. For SNPs with extreme difference in proportion of heterozygotes, we used the BLASTn program to search for sequence alignments using the surrounding sequence of the SNP as provided by dbSNP. Eight of the autosomes with SNPs flagged for sex discrepancy in the NARAC data were further examined in the FHS dataset using the same approaches.

## Results

### NARAC chromosome X

**Figure 1:**
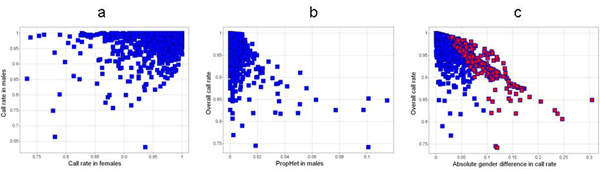
**Plots of sex-specific QC metrics for chromosome X SNPs**. a, Call rate in males vs. females; b, Overall call rate vs. proportion of male heterozygote calls; c, Overall call rate vs. absolute sex difference in call rate.

The call rates for males and females are shown in Figure 1a. Figure 1b plots the overall call rate and the proportion of male heterozygotes. Ideally, one might examine a subset of plots from various portions of the distribution of each QC metric and attempt to choose a threshold beyond which most of the plots confirm a clustering problem. Lacking the intensity data, we chose a call rate difference corresponding to the amount of overall data we would accept for a SNP. For instance, a 5% call rate difference corresponding to a 95% call rate (289 SNPs). There should be no male heterozygote calls. We chose a threshold to flag more than a small number of errant calls given our sample size of 569 males: a 1% threshold flags 76 SNPs. For the missingness test there are 345 SNPs with *p*-values less than 10^-7^. This method flags SNPs with smaller differences between males and females when the overall call rate for the SNP is higher. Figure 1c plots the overall call rate by sex difference in call rate for chromosome X, and highlights in red the SNPs with *p*-values less than 10^-7^.

There are 151, 248, and 21 SNPs flagged exclusively by one, two, or three of these flags. After applying other QC filters of an overall call rate higher than 95% and Hardy-Weinberg *p*-values among controls greater than 10^-6^, there are 108, 90, and 3 of the SNPs that passed these tests had one, two, or three of the sex-difference flags. The three SNPs flagged by all three methods are shown in Table 1.

**Table 1:** Chromosome X SNPs flagged by three sex-specific QC metrics

SNP	Position	Call rate (%)	HWE *p *in female controls	Abs diff call rate (%)	Proportion male heterozygotes (%)	Missing test by sex
rs4893596	2.9 × 10^7^	95	0.144	7.36	1.95	1.69 × 10^-11^
rs7051891	2.9 × 10^7^	95.49	1	1.1	1.4	3.50 × 10^-25^
rs7884174	6.4 × 10^7^	95.83	0.033	9.45	1.18	3.26 × 10^-20^

### NARAC autosomes

**Figure 2:**
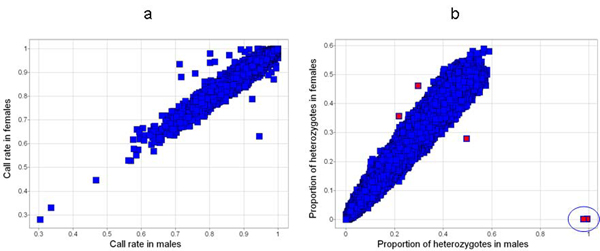
**Plots of sex-specific QC metrics for autosomal SNPs**. a, Call rate in females vs. males; b, Proportion of heterozygotes in females vs. males.

Figure 2a plots the sex-specific call rates for the autosomal SNPs. For 94 SNPs the absolute values of the call rate difference is greater than 5%. Figure 2b plots the proportion of heterozygotes for males and females. In this case, we chose a threshold that would identify the outliers in these data. The six outlier SNPs can be flagged with a difference in the proportion of heterozygotes greater than 13% (highlighted in red). Three are extreme outliers (circled in Figure 2b). These three SNPs are the only SNPs having a sex association more significant than 10^-7^. By utilizing the surrounding sequence for these three SNPs as provided by dbSNP, these three show significant alignment to both chromosomes 1 and Y, resulting in very similar BLASTn E-values and alignment scores [4]. Twenty-three SNPs have a *p*-value less than 10^-7 ^for the test of missingness by sex. There are 96 SNPs flagged by one or more of these thresholds (75, 15, 3, and 3 by one, two, three, and four, respectively). Table 2 lists the 21 SNPs flagged by two or more criteria. After removing SNPs that would be flagged by a 95% overall call rate filter or a HWE filter at 10^-6^, there are three SNPs with two sex-difference flags and three with one flag.

**Table 2:** Autosomal SNPs flagged by at least two sex-specific QC metrics

SNP	Chr	Position	Call rate (%)	HWE *p *in controls	Abs diff call rate (%)	Abs diff in Het (%)	Missing test by sex	Sex association in controls
rs12734338	1	199201380	98.3	2.76 × 10^-12^	5.91	98.87	1.22 × 10^-18^	4.86 × 10^-212^
rs12743401	1	199208305	98.4	1.32 × 10^-11^	5.8	98.98	1.77 × 10^-19^	7.36 × 10^-212^
rs3881953	1	199259678	97.48	2.29 × 10^-11^	8.17	97.5	2.74 × 10^-23^	1.16 × 10^-207^
rs6697552	1	239452393	96.41	0.097	8.39	9.28	2.09 × 10^-17^	0.009
rs7584345	2	67629941	91.22	0.389	8.24	3.88	1.57 × 10^-10^	0.007
rs689662	3	16389202	96.8	0.668	10.63	2.14	3.93 × 10^-31^	0.005
rs17042252	3	16435774	95.15	0.192	5.48	5.08	6.40 × 10^-09^	0.18
rs12508842	4	92374678	90.25	0.068	13.97	11.58	2.69 × 10^-19^	0.005
rs11739167	5	25945521	71.82	1.89 × 10^-6^	31.39	21.74	4.16 × 10^-55^	0.157
rs11155996	6	155056263	85.79	5.30 × 10^-11^	13.87	10.42	1.58 × 10^-14^	0.003
rs10277011	7	64350823	94.81	0.051	5.47	6.75	4.26 × 10^-08^	0.249
rs6989593	8	47670421	83.61	1.53 × 10^-7^	16.44	13.83	7.38 × 10^-18^	0.599
rs2309966	9	71344898	90.93	0.052	12.96	6.61	5.89 × 10^-18^	0.873
rs7046290	9	87432131	93.94	1	7.64	1.08	3.50 × 10^-14^	0.785
rs11034170	11	4819429	82.59	4.96 × 10^-10^	13.61	4.64	1.32 × 10^-14^	0.63
rs7124728	11	70554083	93.06	0.855	17.85	8.24	2.16 × 10^-40^	0.02
rs703842	12	56449006	94.71	0.143	6.57	1.81	4.93 × 10^-12^	0.211
rs17768343	14	37070932	87.34	1.40 × 10^-4^	22.33	16.5	1.30 × 10^-37^	0.176
rs2236225	14	63978598	90.35	4.81 × 10^-4^	11.63	8.9	3.12 × 10^-20^	0.142
rs7294	16	31009822	94.13	0.02	11.56	5.25	1.38 × 10^-20^	0.132
rs6503096	17	8122992	92.34	0.851	7.91	3.78	2.82 × 10^-11^	0.029

### FHS autosomes

We also looked at sex differences on a subset of eight chromosomes from the FHS dataset that were genotyped by Affymetrix 500 k arrays. While there were SNPs (*n *= 51) with sex differences in call rate (>5%) and/or PropHet (>10%) on the chromosomes we examined (Figure 2), all but two of these SNPs would also have been flagged by a 95% call rate or 10^-6 ^HWE filter.

## Discussion

Based on our experience in calling X chromosome data on the Illumina platforms, we expected to find a subset of SNPs where the male-female intensity differences shifted the clusters enough that there would be differences in call rate or some males would be called as heterozygotes. There were outliers for these measures and a test for differential missingness does flag SNPs in the NARAC data that may represent clustering problems. While the possible explanation for sex discrepancies on autosomal SNPs is less clear, we did see a small number of autosomal SNPs as outliers for our sex-specific metrics in the NARAC and FHS data. Most of these are also identified by more traditional QC filters. But a small number would not have been flagged by overall call rate or HWE. Adding sex-specific metrics to the SNP statistics for GWAS studies may allow the original and later investigators to define thresholds to flag these SNPs so that they could be dropped before, or scrutinized more carefully after, analysis. For the tables in the paper we highlighted SNPs with multiple problems. Any one flag may be sufficient to indicate a clustering problem and a need for further review. Knowing there are sex-specific differences with a SNP could aid investigators reviewing a plot of the intensity data. Highlighting males or females while the other sex is colored by genotype can make it much easier to see a sex-specific problem.

Of the five metrics we used, two looked at missingness by sex and two looked at differences in genotype frequencies. The call rate differences by sex and test of missingness are similar, but the test of missingness is affected by the overall call rate for the SNP. Likewise the difference in the proportion of heterozygotes and the test of association with sex look for sex-specific differences in genotype frequencies, with the association test flagging smaller differences when the MAF is low. When the trait is associated with sex, extra care must be taken in applying and interpreting the results of sex-specific filters. Because sex-based confounding is likely to cause at least small differences in allele and genotype frequencies, applying some checks only in the controls may be helpful.

## Conclusion

In the NARAC dataset, we flagged three SNPs on the X chromosome and three on autosomes with sex-specific calling errors that would not have been detected with general QC filters (95% call rate and 10^-6 ^HWE). We flagged two such SNPs in the eight FHS autosomes we examined. Applied with care, sex-specific filters may be useful to identify and filter SNPs before the analysis of GWAS data.

## List of abbreviations used

EIRA: Epidemiologic Investigation of Rheumatoid Arthritis; FHS: Framingham Heart Study; GAW16: Genetic Analysis Workshop 16; GWAS: Genome-wide association study; HWE: Hardy-Weinberg equilibrium; MAF: Minor allele frequency; NARAC: North American Rheumatoid Arthritis Consortium; PropHet: Proportion of heterozygotes; RA: Rheumatoid arthritis; QC: Quality control; SNP: Single-nucleotide polymorphism.

## Competing interests

The authors declare that they have no competing interests.

## Authors' contributions

JEBW analyzed the FHS data. EWP proposed the study, drafted and critically revised the manuscript and suggested further analysis. HL analyzed the NARAC data and drafted the manuscript. KH reviewed results and suggested further analyses. All authors read and approved the final manuscript.
